# Calcaneal Insufficiency Fracture: An Underrecognized Cause of Atraumatic Heel Pain in Older Adults

**DOI:** 10.3390/clinpract16080148

**Published:** 2026-08-13

**Authors:** Koji Nozaka, Naohisa Miyakoshi

**Affiliations:** Department of Orthopaedic Surgery, Akita University Graduate School of Medicine, 1-1-1 Hondo, Akita 010-8543, Japan

**Keywords:** calcaneus, heel pain, insufficiency fracture, osteoporosis, magnetic resonance imaging, older adults

## Abstract

**Background/Objectives**: Calcaneal insufficiency fractures may be overlooked in older adults presenting with atraumatic heel-region pain because early radiographic findings can be absent or inconclusive. Evidence regarding how often these fractures are identified in routine outpatient practice remains limited. This study aimed to describe the observed frequency, diagnostic pathway, clinical characteristics, treatment, and documented clinical course of calcaneal insufficiency fractures in an orthopaedic outpatient cohort. **Methods**: We retrospectively reviewed consecutive patients aged 60 years or older who presented with atraumatic pain localised to the heel or calcaneal region between April 2012 and March 2020. All patients underwent initial plain radiography. Follow-up radiography and MRI were performed selectively when weight-bearing heel pain persisted, radiographic findings remained negative or equivocal, and clinical suspicion of an occult fracture remained. Observed fracture frequencies and exact 95% confidence intervals (CIs) were calculated. **Results**: Among 123 included patients, calcaneal insufficiency fractures were identified in 10, corresponding to an observed frequency of 8.1% (95% CI, 4.0–14.4%). Among 89 independently ambulatory patients, 9 fractures were identified, corresponding to an observed subgroup frequency of 10.1% (95% CI, 4.7–18.3%). These subgroup findings were descriptive and were not adjusted for potential confounders. All 10 fracture cases occurred in women, with a mean age of 78.4 years. Osteoporosis was newly identified in all fracture cases; the mean femoral-neck T-score was −3.42. Four fractures were identified on radiographic findings, whereas six required MRI to establish the diagnosis after radiographs remained negative or inconclusive. All cases were managed non-operatively. The mean interval from symptom onset to the first documented clinical improvement or resolution of heel pain was 28.4 days, and no displacement or subsequent surgical intervention was documented during the available follow-up. **Conclusions**: In this retrospective single-centre cohort, calcaneal insufficiency fractures were identified in a subset of older adults presenting with atraumatic heel-region pain. The observed frequencies should be interpreted as descriptive estimates rather than population prevalence figures. Calcaneal insufficiency fracture may be considered when focal heel pain persists despite normal or equivocal initial radiographs, with repeat radiography and MRI considered according to the clinical context. Prospective studies using standardised imaging and follow-up protocols are needed.

## 1. Introduction

Heel pain is a common reason for presentation to orthopaedic and primary care clinics. Its differential diagnosis is broad and includes plantar fasciitis, Achilles tendon disorders, retrocalcaneal bursitis, heel-pad disorders, peripheral nerve entrapment, inflammatory or infectious conditions, and referred pain [1,2,3]. Because soft-tissue and mechanical disorders account for many cases, atraumatic heel pain is often initially managed without advanced imaging. In older adults, however, skeletal fragility should also be considered as a possible underlying cause.

An insufficiency fracture occurs when physiological loading is applied to bone with reduced mechanical strength, commonly in association with osteoporosis or other conditions affecting bone quality [4]. Such fractures are well recognised in the pelvis, sacrum, and proximal femur, whereas insufficiency fractures involving the calcaneus have been described much less frequently. Published reports specifically addressing calcaneal insufficiency fractures have largely consisted of individual case reports or small case series involving older adults, patients with osteoporosis or inflammatory rheumatic disease, and patients exposed to altered lower-limb loading after total knee arthroplasty [5,6,7,8,9,10]. Broader studies of insufficiency fractures involving the foot, ankle, or adjacent lower-limb regions have also been reported in selected rheumatic disease populations [11,12].

The diagnosis may be difficult during the early clinical course. Initial plain radiographs may be normal or show only subtle changes, whereas delayed radiographs may subsequently demonstrate sclerosis or new bone formation within the calcaneal body. Magnetic resonance imaging (MRI) can demonstrate marrow oedema and a fracture line before these radiographic changes become apparent [13,14,15,16,17,18,19,20,21]. Nevertheless, MRI is not performed systematically in all patients with atraumatic heel pain, and its use in routine practice is generally determined by the persistence and localisation of symptoms, the findings on physical examination, the results of initial radiography, and local resource availability. Consequently, some occult fractures may remain unrecognised.

Available epidemiological evidence remains limited and is derived mainly from selected populations. Calcaneal insufficiency fractures have been investigated after total knee arthroplasty [9,10], whereas MRI-based studies in patients with rheumatic musculoskeletal disease have evaluated insufficiency fractures involving the foot and other lower-limb regions more broadly [11,12]. These populations, anatomical definitions, imaging strategies, and diagnostic denominators differ substantially from those of older adults presenting to a general orthopaedic outpatient clinic with atraumatic heel pain. Accordingly, the frequency with which calcaneal insufficiency fractures are identified in this clinical setting remains uncertain.

The primary aim of this retrospective, single-centre observational study was therefore to describe the observed frequency of calcaneal insufficiency fractures among consecutive patients aged 60 years or older who presented to an orthopaedic outpatient clinic with atraumatic pain localised to the heel or calcaneal region. Secondary aims were to describe the diagnostic pathway, clinical characteristics, treatment, and documented clinical course of the identified fracture cases. Given the retrospective design and non-uniform use of advanced imaging, the study was intended to provide descriptive, hypothesis-generating information rather than a population prevalence estimate or an analysis of independent risk factors.

## 2. Materials and Methods

### 2.1. Study Design and Setting

This was a single-centre retrospective observational study conducted at the orthopaedic outpatient clinic of Akita University Hospital. The study included patients who visited the clinic between 1 April 2012 and 31 March 2020. The clinical records were retrospectively reviewed in 2024.

The study was conducted in accordance with the Declaration of Helsinki and was approved by the Ethics Committee of Akita University Graduate School of Medicine (approval No. 1970). The requirement for individual informed consent was waived because of the retrospective nature of the study and the use of anonymised clinical data.

### 2.2. Participants

We included consecutive patients aged 60 years or older who presented with atraumatic pain clinically localised to the heel or calcaneal region and underwent plain radiographic assessment of the calcaneus during the study period.

Patients with an obvious traumatic injury or another clearly identifiable acute traumatic condition affecting the heel or hindfoot at presentation were excluded. The eligibility criterion was intentionally broad because the study aimed to describe the observed frequency of calcaneal insufficiency fractures among older adults presenting with atraumatic heel-region pain in routine outpatient practice, rather than among patients in whom a fracture was already strongly suspected.

The final study cohort comprised 123 patients, including 46 men and 77 women. At presentation, 89 patients were independently ambulatory, whereas 33 used a wheelchair and one arrived on a stretcher. Because this was a retrospective study based on routine outpatient documentation, detailed baseline information, including age, symptom duration, and osteoporosis status, was not consistently available for all patients in whom no calcaneal insufficiency fracture was identified.

### 2.3. Initial Clinical and Radiographic Assessment

All 123 patients underwent plain radiography at the initial outpatient visit. Initial clinical assessment was performed according to routine practice and included evaluation of pain location, pain during weight-bearing, and local tenderness.

Because this was a retrospective study, the indications for additional imaging were not defined by a prospectively standardised protocol. When the initial radiographs were normal or equivocal, follow-up radiography was generally considered approximately 2 weeks later if weight-bearing heel pain persisted despite initial conservative management.

Clinical suspicion of calcaneal insufficiency fracture was considered greater when marked focal tenderness was present over the calcaneal body, including its medial or lateral aspect, rather than being confined to the Achilles tendon insertion or plantar fascia origin. These findings informed, but did not independently determine, the treating physician’s decision to obtain additional imaging.

### 2.4. Follow-Up Imaging and Diagnostic Criteria

Follow-up radiography was performed in 26 patients, generally approximately 2 weeks after the initial assessment because heel pain persisted. The same 26 patients subsequently underwent MRI when follow-up radiographs remained negative or inconclusive and clinical suspicion of calcaneal insufficiency fracture persisted. The remaining 97 patients underwent neither follow-up radiography nor MRI.

In patients who did not undergo additional imaging, no calcaneal insufficiency fracture was identified during the available clinical follow-up. However, because MRI was not performed systematically in these patients, occult fracture could not be definitively excluded.

MRI was performed using a 1.5-T system with an extremity coil. The routine protocol included axial, sagittal, and coronal T1-weighted sequences and fat-suppressed T2-weighted sequences.

Calcaneal insufficiency fracture was diagnosed when imaging findings were considered compatible with a fracture in the absence of obvious trauma, repetitive overuse, or another apparent precipitating event. On radiography, delayed osteosclerotic change or new bone formation within the calcaneal body was considered supportive of the diagnosis. On MRI, the diagnosis was based on a linear low-signal-intensity lesion within the calcaneus accompanied by surrounding marrow oedema, characterised by low signal intensity on T1-weighted images and high signal intensity on fat-suppressed T2-weighted images.

### 2.5. Bone Mineral Density Assessment

In the 10 patients in whom calcaneal insufficiency fracture was identified, bone mineral density was assessed at the femoral neck using dual-energy X-ray absorptiometry (DXA; QDR 4500A, Hologic, Waltham, MA, USA). DXA was performed within 4 weeks of the initial presentation as part of the clinical evaluation for underlying skeletal fragility.

Femoral-neck T-scores were obtained for all 10 fracture cases. The medical records were also reviewed to determine whether osteoporosis had been diagnosed or treated before the diagnosis of calcaneal insufficiency fracture. Bone mineral density was not assessed systematically in patients without identified fractures; therefore, the study was not designed to compare osteoporosis prevalence between patients with and without fractures.

### 2.6. Treatment and Follow-Up Assessment

Treatment was selected according to the treating physician’s clinical judgement and was not based on a prospectively standardised protocol. Initial weight-bearing restriction consisted of non-weight-bearing or, when strict non-weight-bearing was impractical, the minimum feasible level of protected weight-bearing, generally toe-touch weight-bearing.

A below-knee cast maintaining the ankle in a neutral position was generally used when heel pain was pronounced and greater immobilisation was considered clinically appropriate. Patients for whom cast immobilisation was considered less appropriate because of their general condition or mobility requirements were managed with weight-bearing restriction alone.

Clinical outcome was assessed retrospectively from routine outpatient medical records, primarily on the basis of the treating physician’s documentation of patient-reported changes in heel pain. No prospectively standardised follow-up protocol or validated patient-reported outcome measure was used. The clinical outcome was therefore defined as the first documented clinical improvement or resolution of heel pain.

Follow-up duration was calculated from the initial outpatient assessment to the most recent available clinical evaluation. The occurrence of fracture displacement, additional immobilisation, and surgical intervention was also recorded.

### 2.7. Outcome Measures

The primary outcome was the observed frequency of calcaneal insufficiency fractures among the included patients with atraumatic heel-region pain.

Secondary descriptive outcomes included the observed frequency among independently ambulatory patients, the imaging pathway leading to fracture identification, age and sex of the fracture cases, interval from symptom onset to the initial visit, bone mineral density findings, treatment modality, interval from symptom onset to the first documented clinical improvement or resolution of heel pain, follow-up duration, fracture displacement, and subsequent surgical intervention.

The independently ambulatory and non-ambulatory groups were described as clinical subgroups. No inference regarding an independent association between ambulatory status and fracture occurrence was intended because potential confounding factors were not adjusted for.

### 2.8. Statistical Analysis

Given the exploratory and descriptive nature of this retrospective study and the small number of identified fracture cases, no formal hypothesis testing or multivariable analysis was performed.

Continuous variables are presented as means with ranges when available. Categorical variables are presented as counts and percentages. Ninety-five percent confidence intervals for the observed fracture frequencies were calculated using the exact Clopper–Pearson method.

Missing data were not imputed. Variables that were not documented consistently in the available retrospective records were not included in formal comparisons.

## 3. Results

### 3.1. Study Cohort and Observed Fracture Frequency

A total of 123 patients aged 60 years or older who presented with atraumatic heel-region pain met the eligibility criteria and were included in the study. The cohort comprised 46 men and 77 women. At presentation, 89 patients were independently ambulatory, whereas 33 used a wheelchair and one arrived on a stretcher.

Calcaneal insufficiency fractures were identified in 10 of the 123 included patients, corresponding to an observed frequency of 8.1% (95% confidence interval [CI], 4.0–14.4%). Of the 123 patients, 89 were independently ambulatory; calcaneal insufficiency fractures were identified in 9 of these patients, corresponding to an observed frequency of 10.1% (95% CI, 4.7–18.3%) within this subgroup. Among the 34 patients who required wheelchair or stretcher assistance, one fracture was identified, corresponding to an observed frequency of 2.9% (95% CI, 0.1–15.3%). These subgroup frequencies are presented descriptively; no adjusted comparison was performed.

Among the 113 patients in whom no calcaneal insufficiency fracture was identified, 46 were men and 67 were women; 80 were independently ambulatory at presentation, whereas 33 required wheelchair or stretcher assistance. Age, symptom duration, and osteoporosis-related information were not consistently available for this group and were therefore not formally summarised or compared (Table 1).

**Table 1:** Descriptive characteristics of the study cohort and identified fracture cases.

Characteristic	Overall Cohort (*n* = 123)	Identified Fracture Cases (*n* = 10)	No Identified Fracture (*n* = 113)
Number of patients	123	10	113
Sex, *n* (%)			
Female	77 (62.6)	10 (100)	67 (59.3)
Male	46 (37.4)	0 (0)	46 (40.7)
Mobility at presentation, *n* (%)			
Independently ambulatory	89 (72.4)	9 (90.0)	80 (70.8)
Wheelchair or stretcher assistance	34 (27.6)	1 (10.0)	33 (29.2)
Age, years, mean (range)	Not consistently available	78.4 (69–88)	Not consistently available
Body mass index	Not consistently available	Not reliably available	Not consistently available
Femoral-neck T-score, mean (range)	Not systematically assessed	−3.42 (−3.9 to −2.9)	Not systematically assessed
Previously diagnosed osteoporosis, *n* (%)	Not consistently available	0 (0)	Not consistently available
Prior pharmacological osteoporosis treatment, *n* (%)	Not consistently available	0 (0)	Not consistently available

Data are presented descriptively. Age, body mass index, and osteoporosis-related information were not consistently available for patients in whom no calcaneal insufficiency fracture was identified. Bone mineral density was assessed systematically only in the 10 identified fracture cases. No formal statistical comparison between groups was performed.

### 3.2. Clinical Characteristics and Bone Mineral Density of the Fracture Cases

All 10 patients in whom calcaneal insufficiency fractures were identified were women. Their mean age was 78.4 years (range, 69–88 years). None had a clearly documented traumatic event, repetitive overuse episode, or other apparent precipitating event before symptom onset.

No patient had a documented history of rheumatoid arthritis, diabetes mellitus, systemic corticosteroid use, or another clearly recorded secondary cause of skeletal fragility in the available medical records. Body mass index could not be calculated reliably because height and weight were not systematically documented in the retrospective outpatient records.

Osteoporosis was newly identified during the post-diagnostic evaluation in all 10 fracture cases. None of the patients had previously received a diagnosis of osteoporosis or pharmacological osteoporosis treatment. Femoral-neck bone mineral density was assessed by dual-energy X-ray absorptiometry in all 10 patients. The mean T-score was −3.42 (range, −3.9 to −2.9). After confirming the absence of contraindications, oral bisphosphonate therapy was initiated after the diagnosis of calcaneal insufficiency fracture and continued during follow-up in all 10 patients.

Because bone mineral density was not assessed systematically in patients without identified fractures, no comparison of osteoporosis status between the fracture and non-fracture groups was performed.

### 3.3. Imaging Pathway

All 123 patients underwent plain radiography at the initial outpatient visit. Follow-up radiography was subsequently performed in 26 patients, generally approximately 2 weeks after the initial assessment because heel pain persisted despite initial management. The same 26 patients underwent MRI when follow-up radiographs remained negative or inconclusive and clinical suspicion of calcaneal insufficiency fracture persisted. The remaining 97 patients underwent neither follow-up radiography nor MRI.

**Figure 1:**
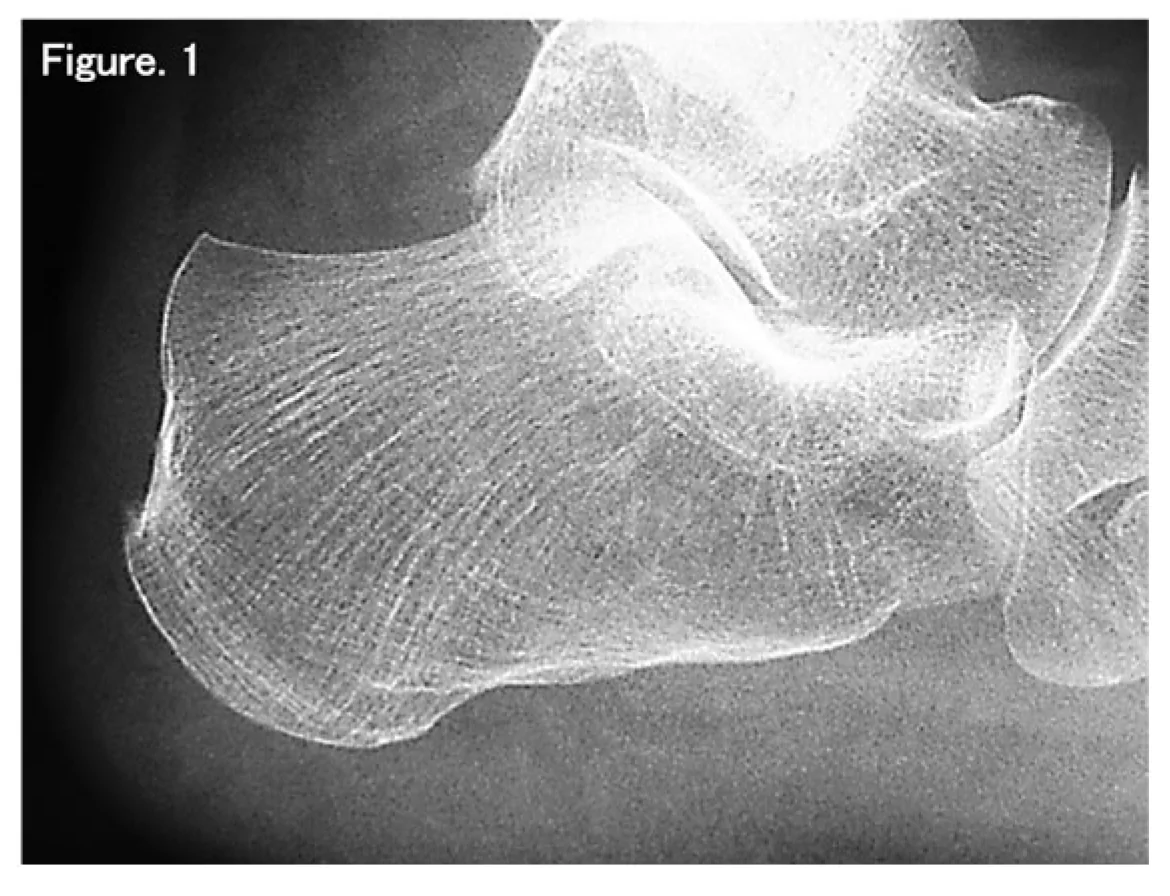
Initial radiograph of a 77-year-old woman. No definite fracture line is visible at the first visit despite atraumatic heel pain.

**Figure 2:**
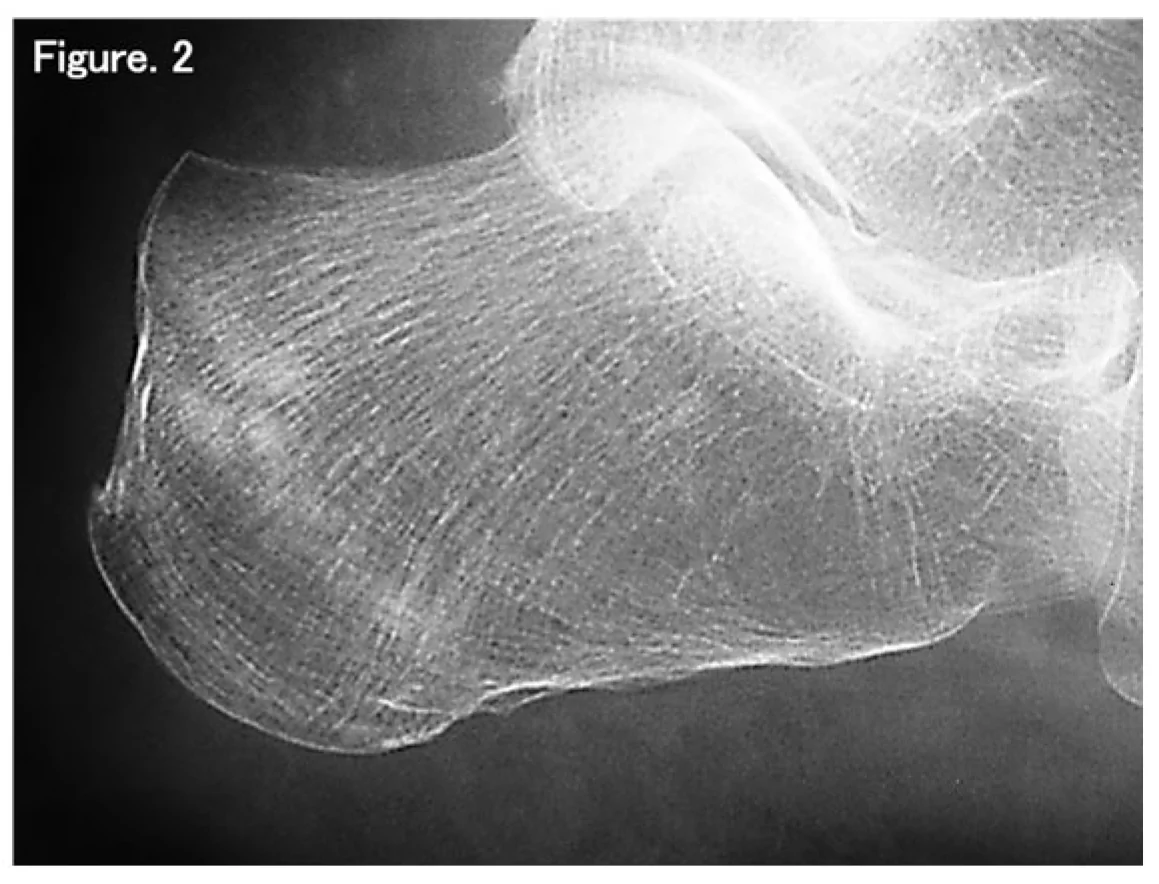
Follow-up radiograph of the same 77-year-old woman. Osteosclerotic change became apparent 2 weeks after the initial visit, supporting the diagnosis of calcaneal insufficiency fracture.

**Figure 3:**
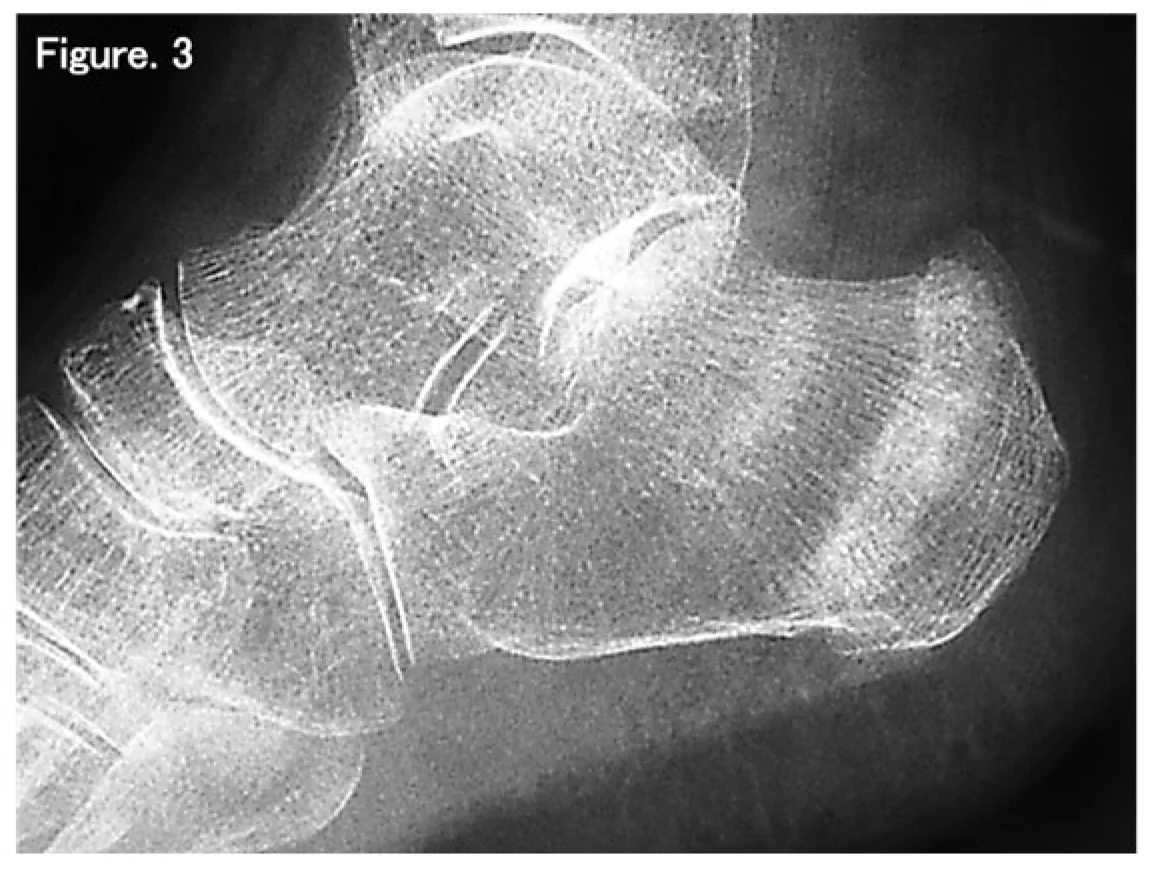
Initial radiograph of a 74-year-old woman who presented 5 weeks after symptom onset. Osteosclerotic change is already visible at the first visit.

**Figure 4:**
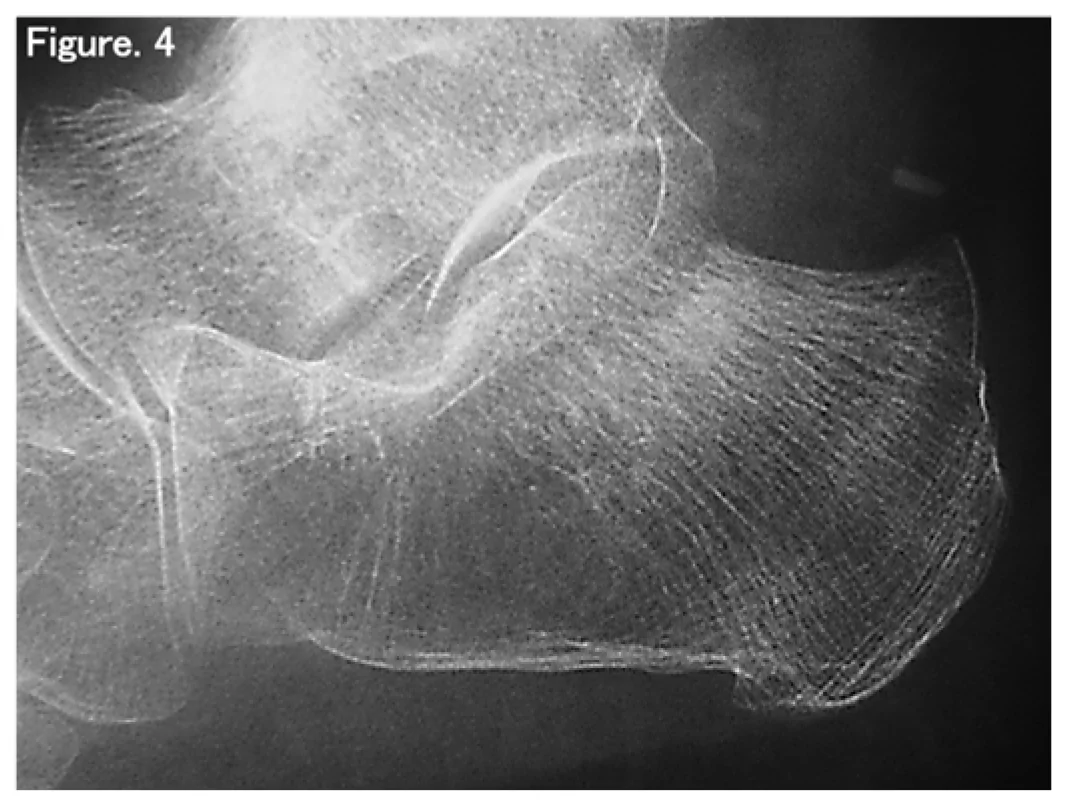
Follow-up radiograph of the same 74-year-old woman. Osteosclerotic change resolved during follow-up after conservative treatment.

**Figure 5:**
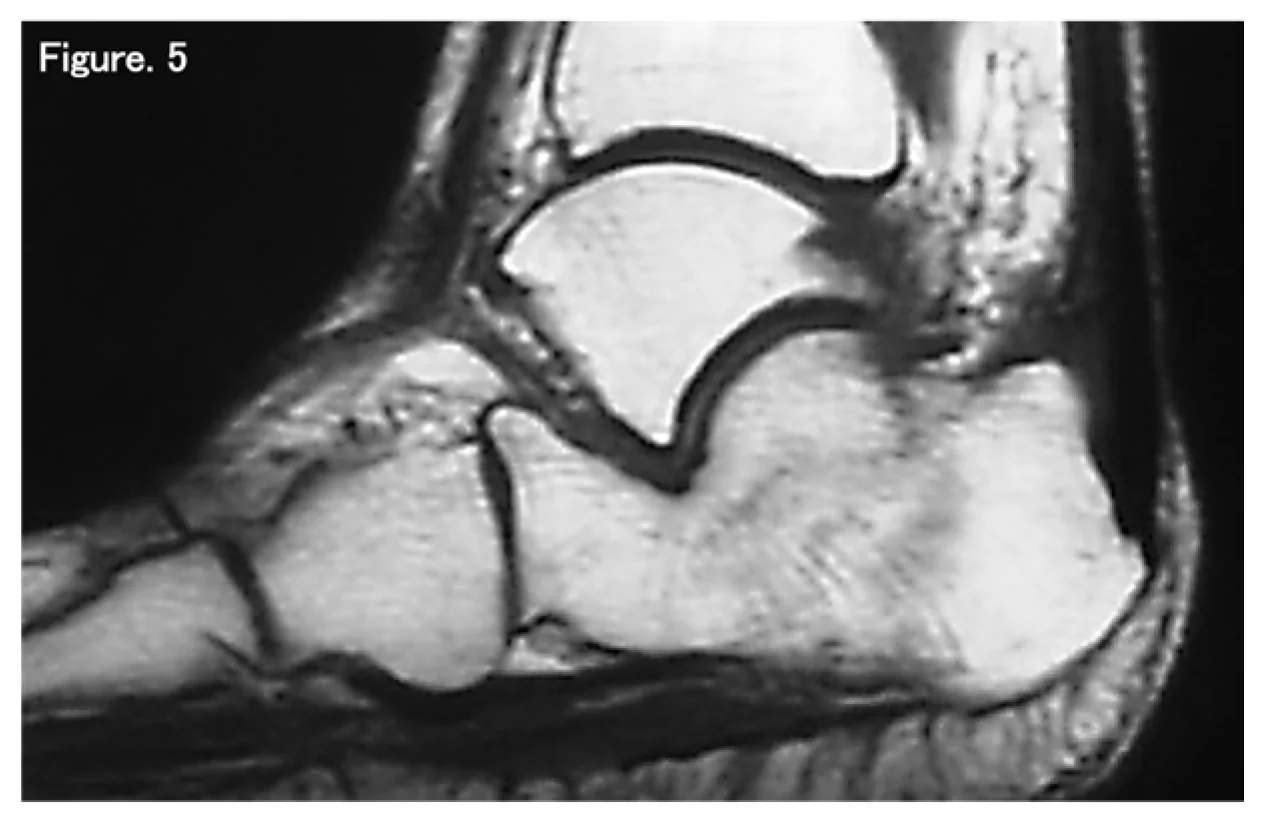
Sagittal T1-weighted MRI of an 86-year-old woman showing a linear low-signal lesion in the calcaneus.

**Figure 6:**
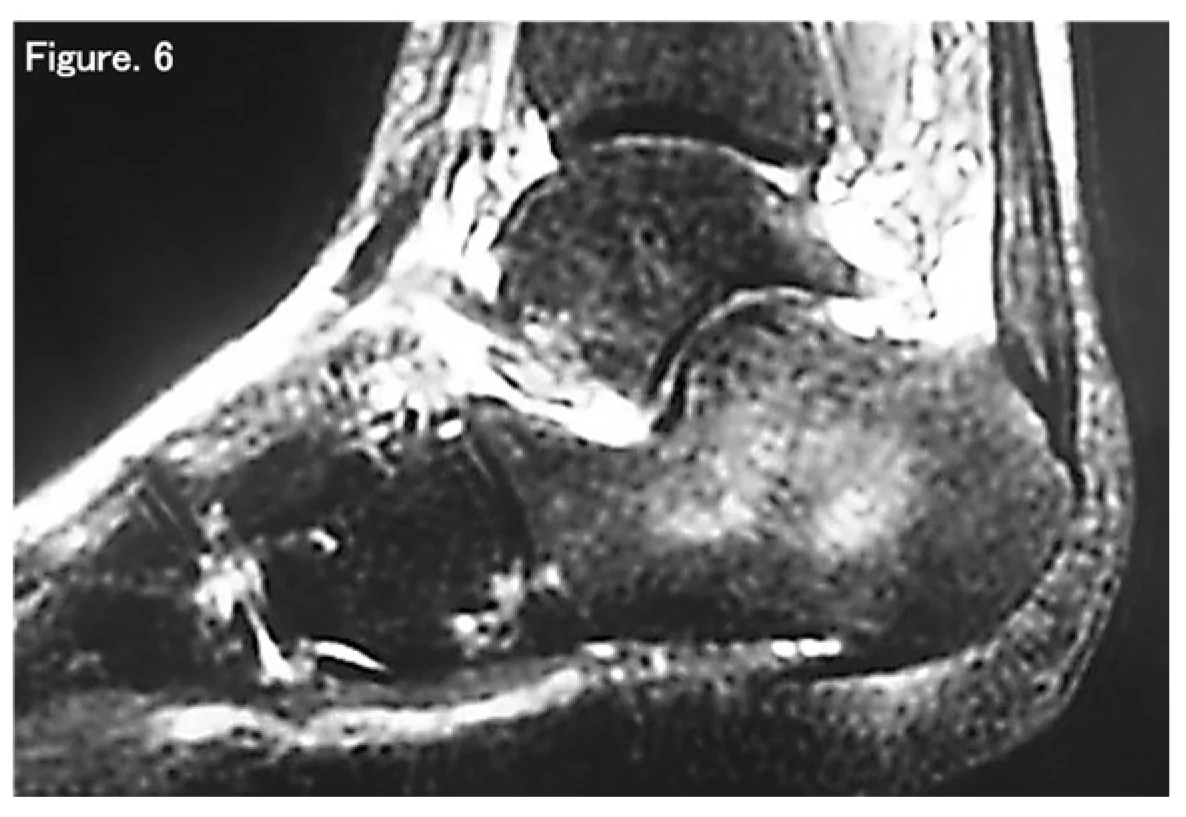
Sagittal fat-suppressed T2-weighted MRI of the same patient showing corresponding high-signal marrow oedema consistent with calcaneal insufficiency fracture.

Among the 10 identified fracture cases, four were diagnosed on radiographic findings without requiring MRI for diagnostic confirmation (Figure 1, Figure 2, Figure 3 and Figure 4). In two of these four patients, osteosclerotic changes were visible on the initial radiographs because they presented relatively late after symptom onset. In the remaining six fracture cases, initial and follow-up radiographs were negative or inconclusive, and MRI findings were required to establish the diagnosis (Figure 5 and Figure 6).

The mean interval from symptom onset to the initial outpatient visit among the fracture cases was 17.4 days (range, 0–40 days).

Among the 97 patients who did not undergo additional imaging, no calcaneal insufficiency fracture was identified during the available clinical follow-up. However, occult fracture was not definitively excluded by MRI in these patients.

### 3.4. Treatment and Documented Clinical Course

All 10 fracture cases were managed non-operatively on an outpatient basis. Eight patients were treated with a below-knee cast maintaining the ankle in a neutral position. The mean duration of cast immobilisation was 21.5 days (range, 12–28 days). The remaining two patients were managed without casting.

Initial weight-bearing restriction consisted of non-weight-bearing or, when strict non-weight-bearing was considered impractical, toe-touch weight-bearing. Treatment selection was based on the treating physician’s clinical judgement. Casting was generally used when heel pain was pronounced and greater immobilisation was considered clinically appropriate.

The mean interval from symptom onset to the first documented clinical improvement or resolution of heel pain was 28.4 days (range, 24–51 days). This assessment was based on routine clinical documentation rather than on a standardised outcome measure.

The mean available clinical follow-up was 47.2 months (range, 12–80 months). No fracture displacement or subsequent surgical intervention was documented during the available follow-up (Table 2).

**Table 2:** Imaging pathway, treatment, and documented clinical course of the identified fracture cases.

Characteristic	Value
Imaging pathway in the overall cohort	
Initial plain radiography, *n*/*N* (%)	123/123 (100)
Follow-up radiography, *n*/*N* (%)	26/123 (21.1)
MRI after negative or inconclusive follow-up radiography, *n*/*N* (%)	26/123 (21.1)
No follow-up radiography or MRI, *n*/*N* (%)	97/123 (78.9)
Diagnostic findings in the identified fracture cases (*n* = 10)	
Diagnosed on radiographic findings without MRI confirmation, *n* (%)	4 (40.0)
MRI required to establish the diagnosis, *n* (%)	6 (60.0)
Interval from symptom onset to initial outpatient visit, days, mean (range)	17.4 (0–40)
Treatment and documented clinical course of the fracture cases (*n* = 10)	
Managed non-operatively, *n* (%)	10 (100)
Below-knee cast immobilisation, *n* (%)	8 (80.0)
Managed without cast immobilisation, *n* (%)	2 (20.0)
Duration of cast immobilisation, days, mean (range)	21.5 (12–28)
Initial non-weight-bearing or toe-touch weight-bearing, *n* (%)	10 (100)
Interval from symptom onset to first documented clinical improvement or resolution of heel pain, days, mean (range)	28.4 (24–51)
Available clinical follow-up, months, mean (range)	47.2 (12–80)
Fracture displacement during available follow-up, *n* (%)	0 (0)
Subsequent surgical intervention, *n* (%)	0 (0)

MRI, magnetic resonance imaging. Treatment was selected according to the treating physician’s clinical judgement and was not prospectively standardised. Clinical improvement was determined retrospectively from routine medical-record documentation rather than from a validated outcome measure.

## 4. Discussion

In this retrospective single-centre outpatient cohort, calcaneal insufficiency fractures were identified in 10 of 123 older patients presenting with atraumatic heel-region pain, corresponding to an observed frequency of 8.1%. Among the 89 independently ambulatory patients, fractures were identified in 9 patients, corresponding to an observed subgroup frequency of 10.1%. These values should be interpreted as descriptive estimates within this selected clinical cohort rather than as population prevalence estimates. In particular, the independently ambulatory and non-ambulatory groups were not adjusted for differences in age, sex, comorbidities, disease severity, or referral patterns; therefore, the present findings do not establish ambulatory status as an independent factor associated with fracture occurrence. Published evidence regarding the epidemiology of calcaneal insufficiency fractures remains limited. Most previous publications have consisted of case reports or small case series involving older women, patients with osteoporosis or inflammatory rheumatic disease, or patients exposed to altered lower-limb loading after total knee arthroplasty [5,6,7,8,9,10,11,12]. Alonso-Bartolome et al. described seven calcaneal insufficiency fractures in six women with a mean age of 73.8 years; initial radiographs were normal in five patients, and MRI was used to establish the diagnosis [6]. In selected postoperative populations, Yamamoto et al. identified 17 fractures among 3585 patients after total knee arthroplasty (0.5%), whereas Kato et al. reported nine fractures after 1548 total knee arthroplasties (0.58%) [9,10]. These studies involved a substantially different denominator and a specific postoperative change in lower-limb loading; therefore, their estimates are not directly comparable with the observed frequency in the present outpatient heel-pain cohort. Evidence from broader high-risk populations also suggests that insufficiency fractures of the foot may be clinically under-recognised. In an MRI-based study of patients with rheumatic musculoskeletal disease and foot pain, Buehring et al. identified foot insufficiency fractures in 7.5% of MRI examinations, whereas conventional radiography identified only 25% of those fractures [11]. This study included multiple anatomical locations within the foot and a selected rheumatology population, and its findings therefore cannot be directly compared numerically with the present cohort. Nevertheless, it illustrates that insufficiency fractures may be encountered among patients with foot pain and may remain occult on plain radiographs. Similarly, a case-series and case–control study of patients with rheumatoid arthritis reported insufficiency fractures of the knee, ankle, and foot across a wide range of bone mineral density values, indicating that these fractures are not confined exclusively to patients meeting densitometric criteria for osteoporosis [12]. The present findings also illustrate the limitations of a single initial radiographic examination. Six of the 10 identified fracture cases required MRI findings to establish the diagnosis because radiographs remained negative or inconclusive. Previous reports have similarly described normal initial radiographs in many patients with calcaneal insufficiency fractures [6,10]. However, this observation should not be interpreted as supporting immediate MRI for every older patient with heel pain. In routine practice, the need for additional imaging should be considered in relation to the location and persistence of pain, physical examination findings, initial radiographic findings, patient mobility, and local availability of imaging resources. Within the limitations of the present retrospective study, a staged diagnostic approach may be considered when initial radiographs are normal or equivocal. In the present cohort, follow-up radiography and MRI were generally considered when weight-bearing heel pain persisted for approximately 2 weeks despite initial management and when marked focal tenderness remained over the calcaneal body rather than being confined to the Achilles tendon insertion or plantar fascia origin. During this interval, temporary immobilisation, protected weight-bearing, and clinical reassessment may be appropriate according to the individual clinical situation. MRI may then be considered when focal symptoms persist, repeat radiographs remain inconclusive, and suspicion of an occult fracture remains. This proposed approach reflects the institutional clinical experience at our centre and should be regarded as a description of the clinical pathway used in the present retrospective cohort rather than as a prospectively validated diagnostic algorithm. Its applicability to other clinical settings remains uncertain and should be evaluated in future prospective multicentre studies. High-resolution musculoskeletal ultrasonography may provide additional information during the initial evaluation of heel pain, particularly when plantar fascia, Achilles tendon, or other soft-tissue disorders are being considered. Ultrasonography was performed in some patients in the present cohort, but it was not used systematically and was not performed specifically to diagnose or exclude calcaneal insufficiency fractures. A previous series described eight calcaneal stress fractures detected using sonography, with findings including periosteal thickening, subcutaneous oedema, cortical irregularity, and local hypervascularity [22]. Nevertheless, the available evidence is limited, the reported population included stress fractures rather than specifically osteoporotic insufficiency fractures, and MRI remained an important confirmatory modality. Accordingly, the possible role of ultrasonography in the diagnostic pathway for calcaneal insufficiency fracture requires further prospective evaluation. Osteoporosis was newly identified during post-diagnostic assessment in all 10 fracture cases, with a mean femoral-neck T-score of −3.42. This observation is clinically relevant because the diagnosis of an insufficiency fracture may provide an opportunity to assess previously unrecognised skeletal fragility. However, bone mineral density was not assessed systematically in patients in whom no fracture was identified. The present study therefore cannot determine whether osteoporosis was more common among fracture cases than among the remainder of the cohort, nor can it establish osteoporosis as an independent or causal risk factor for calcaneal insufficiency fracture. The bone mineral density findings should consequently be interpreted as a descriptive clinical characteristic of the fracture cases. Nevertheless, when a calcaneal insufficiency fracture is suspected or identified, assessment for underlying osteoporosis may be considered as part of the broader clinical evaluation. All 10 identified fracture cases were managed non-operatively, using below-knee cast immobilisation or weight-bearing restriction according to the treating physician’s clinical judgement. Documented clinical improvement or resolution of heel pain occurred after a mean of 28.4 days from symptom onset, and no displacement or subsequent surgical intervention was documented during the available follow-up. Previous small series have also reported symptom improvement following conservative treatment [6,9,10]. However, the present observations do not establish the efficacy or superiority of conservative treatment. Treatment selection was not standardised, only 10 fracture cases were included, there was no comparison group, and no validated patient-reported or functional outcome measure was used. These findings should therefore be regarded as a descriptive account of the clinical course rather than evidence supporting a specific treatment regimen.

## 5. Limitations

This study has several methodological limitations. First, its retrospective single-centre design and the small number of identified fracture cases limit generalisability. Second, repeat radiography and MRI were performed selectively rather than systematically in all patients with negative or equivocal initial radiographs. Patients who did not undergo MRI cannot be considered to have had an occult fracture definitively excluded. This verification bias may have resulted in missed fractures and underestimation of the observed frequency. In addition, because MRI was preferentially performed in patients with persistent symptoms and continued clinical suspicion of calcaneal insufficiency fracture, selection (spectrum) bias may also have occurred. Patients with milder symptoms or spontaneous clinical improvement may therefore have remained undiagnosed. Third, baseline information, including age, symptom duration, body mass index, and osteoporosis-related data, was incomplete for many patients in whom no fracture was identified, precluding reliable comparison between fracture and non-fracture groups. Fourth, the analysis of ambulatory status was descriptive and was not adjusted for potential confounding factors. Fifth, treatment and follow-up assessments were not prospectively standardised, and clinical outcome was derived from routine medical-record documentation rather than a validated outcome instrument. Within these limitations, the present study provides a descriptive estimate of how often calcaneal insufficiency fractures were identified in one orthopaedic outpatient cohort of older adults with atraumatic heel-region pain. The findings do not establish population prevalence, independent risk factors, or treatment efficacy. They do, however, suggest the clinical relevance of including calcaneal insufficiency fracture in the differential diagnosis when focal heel pain persists and initial radiographs are negative or inconclusive. Prospective multicentre studies using standardised clinical criteria, systematic follow-up imaging, complete baseline bone-health assessment, and validated clinical outcome measures are needed to clarify the frequency, associated factors, diagnostic pathway, and clinical course of this condition.

## 6. Conclusions

In this retrospective single-centre outpatient cohort, calcaneal insufficiency fractures were identified in a subset of older adults presenting with atraumatic heel-region pain. The observed frequency was 8.1% in the overall cohort and 10.1% among independently ambulatory patients; however, these values should be interpreted as descriptive estimates rather than population prevalence figures.

Calcaneal insufficiency fracture may be considered in the differential diagnosis when focal heel pain persists despite normal or equivocal initial radiographs. In such cases, repeat radiography and, when clinical suspicion remains, MRI may be considered according to the individual clinical context and availability of imaging resources.

The diagnosis may also provide an opportunity to assess underlying skeletal fragility. However, the present findings do not establish osteoporosis as an independent risk factor or demonstrate the efficacy of a specific treatment strategy. Further prospective multicentre studies using standardised diagnostic and follow-up protocols are warranted.

## Data Availability

The data presented in this study are available on request from the corresponding author, subject to institutional and ethical restrictions.

## Abbreviations

MRI	magnetic resonance imaging
